# The evolutionary trajectory of mitochondrial carrier family during metazoan evolution

**DOI:** 10.1186/1471-2148-10-282

**Published:** 2010-09-16

**Authors:** Ming Gong, Jie Li, Meng Wang, Jin Wang, Ke Zen, Chen-Yu Zhang

**Affiliations:** 1 Jiangsu Diabetes Center, State Key Laboratory of Pharmaceutical Biotechnology, School of Life Sciences, Nanjing University, 22 Hankou Road, Nanjing, Jiangsu 210093, China

## Abstract

**Background:**

Exploring metabolic evolution is a way to understand metabolic complexity. The substrate transport of mitochondrial carrier family (MCF) influences direct metabolic activities, making it possible to understand indirectly metabolic evolution from the evolution of substrate transport of MCF. However, the evolutionary study of substrate transport of MCF does not mean that all the concrete structures of mitochondrial carriers (MCs) must first be gained.

**Results:**

Here we studied the alternation of MCF structure and potential correlated functions of MCF during metazoan evolution. The data analysis indicates that the types of substrates transported by MCF as a whole were maintained during metazoan evolution. However, the size of the substrates transported by members of MCs continuously diminished during the evolutionary process. We have found that the ratio of hydrophobic amino acids at specific helix-helix interfaces increases significantly during vertebrate evolution. Amino acid's spatial positioning and the calculating of packing values both indicate the increase in the number of hydrophobic amino acids would lead to a more "tight" structure of the TR domain, which is in agreement with the trend of diminishing size of substrates transported by MCs. In addition, there was a significant increase in the number of carriers of MCF during vertebrate evolution.

**Conclusions:**

We propose that the more "tight" TR structure generated by the increase of the hydrophobic amino acids at specific helix-helix interfaces during vertebrate evolution enhances the substrate selectivity of MCF, reflecting the evolutionary trajectory of MCF during metazoan evolution.

## Background

Mitochondrial carriers (MCs), located at the inner membrane of mitochondria (IMM), are responsible for transporting a variety of metabolites between the cytosol and the mitochondria and influence the activities of the metabolic pathway direct [1,2]. The substrates transported by MCs, such as nucleotides, amino acids, cofactors, and H^+^, are involved in oxidative phosphorylation, gluconeogenesis, the synthesis and degradations of amino and fatty acids, the macromolecular synthesis of proteins and nucleic acids, sterol metabolism, and thermogenesis [1]. The mutations of the carriers are closely correlated with diseases, including Amish microcephaly, CAC deficiency, NICCD/CTLN2, neonatal myoclonic epilepsy, Sengers' syndrome, severe obesity, and type II diabetes [1,3].

The substrates transported by MCs vary widely in their structure and size from the smallest, H^+^, to the largest and most highly charged species, such as ATP. By considering conservation of amino acids, distance and chemical constraints [4], and by modelling family members [5-12] on the known structure of the ADP/ATP carrier, recent research has identified a common substrate binding site located at approximately the midpoint of the membrane [4]. However, the molecular mechanism of substrate selectivity of MCs remains incompletely understood. It is not possible to obtain the concrete structures of all mitochondrial carrier family (MCF) members through crystallization [1,13], and thus uncovering the mechanism of substrate selectivity of MCF direct is impossible solely by linear superposition.

A powerful source of functional innovation is gene duplication and loss [14]. Some reports have suggested that many of gene duplication events occur during the earlier period of vertebrate evolution [15,16], indicating that if gene duplications exist in MCF [17,18], the substrate selectivity changes may be a form of functional innovation closely associated with metabolic evolution. Here we investigate MCF, a gene family with common functional categories whose members do not have clearly defined functions, and subject it to The Big Experiment [19] to explore the evolutionary trajectory of MCF. We further investigate the synchronization between molecular evolution and macroevolution to search for evolutionary rules of sequences corresponding to structures of MCF in order to determine the characteristics of structural changes. The molecular mechanism of change of substrate selectivity was then analyzed to identify the transport functions of MCs.

## Results

### Gene duplication of MCF is under strong purifying selection

According to the classification of the MCF secondary structure region (Additional file 1), TR has the most conservative sequence with an identity of 70.44%, while the average identities of LOOP135 (loop1, loop3, loop5) and NCLOOP24 (n, c, loop2, loop4) (Additional file 1) are only 42.24% and 19.25%, respectively. On the one hand, the high evolutionary conservation in TR reflects a stable structure maintaining the basic function of the transport channel. On the other hand, this evolutionary conservation indicates that the relatively small changes (29.56%) of TR sequences may have a large effect on the substrate transport of MCF.

**Figure 1:**
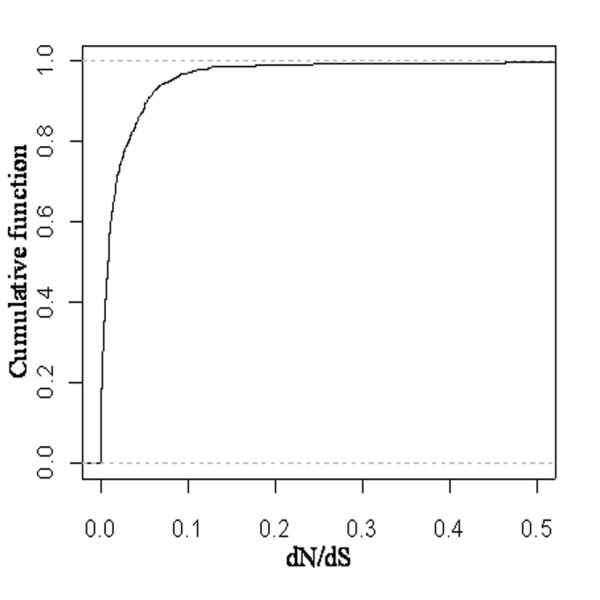
**The cumulative distribution of *d_N _/d_S _*of MCF**. The orthologous sequences of SLC25 were applied into a pairwise sequence comparison. The program yn00 used the methods of Yang and Nielsen [50] to estimate synonymous and nonsynonymous substitution rates between two orthologous sequences (*d*_S _and *d*_N_).

Based on phylogenetic trees constructed from TR sequences, orthologous subfamilies were identified from gene families along with the relative gene duplication events. Fifteen gene duplication events were found in twelve gene families (Additional file 2). For the gene family tested, the alternative model (specifically testing for positive selection) was not a significantly better fit (2λ < 2.71, df = 1, p > 0.05) than the null model (Additional file 3). This result shows that there is no positive selection in the evolution of MCF. According to purifying selection (*d_N _/d_S _*< 1), the smaller the *d_N _/d_S _*ratio is, the greater the number of eliminated substitutions and the greater the selective constraint [20]. Therefore, strong purifying selection is characteristic of a high number of eliminated substitutions and a strong selective constraint, indicating that its *d_N_/d_S _*values must be very small. The cumulative distribution of the *d_N _/d_S _*of MCF (Figure 1) and the mean value of *d_N _/d_S _*(0.023) both show that strong purifying selection occurs in the evolution of MCF in metazoan. The strong purifying selection indicates that MCF maintains a relatively stable substrate transport and its evolution is characteristic of continuity during metazoan evolution.

### The evolution of MCF keeps pace with metazoan evolution

**Figure 2:**
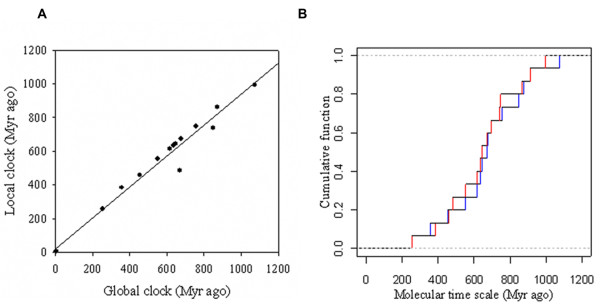
**Age distribution of gene duplication events of MCF**. (*A*) The linear regression of the duplication time estimates between global clock and local clock. The coefficient of correlation is 0.977 (p < 0.001). (*B*) Distribution of the age distribution estimated by global clock and local clock. Red indicates the age estimated by a local clock. Blue indicates the age estimated by the global clock. The molecular time scale was measured as Myr ago.

The distribution of gene duplication events was found mainly 750-430 Myr ago (Figure 2A), meeting approximately the earlier stage of vertebrate evolution along with a large scale of events of gene duplication [15,16]. According to the trend of the cumulative distribution of age distribution (Figure 2B), an explosion of gene duplication events occurred approximately 600 Myr ago during the Ediacarian and Cambrian periods (approximately 630 - 490 Myr ago). This timing indicates that the functional evolution of MCF began at approximately the same time as metazoan evolution.

**Figure 3:**
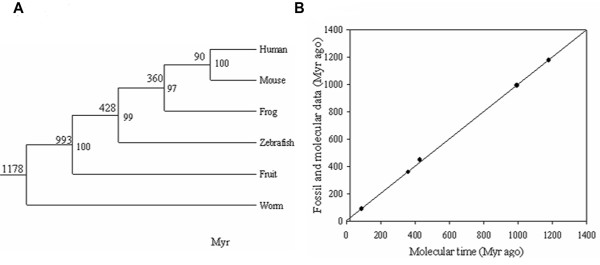
**Analysis of the synchronization between the molecular evolution of MCF and macroevolution**. (*A*) The molecular clock estimation of metazoan evolution based on six tandem concatenated TR sequence (see methods). The phylogenetic tree was inferred by the neighbor-joining method [43] (bootstrap = 1000, JTT matrix). (*B*) The linear regression of diversification time estimates between the local clock estimation and that inferred from the fossil and molecular data [52,53]. The coefficient of correlation is 0.99 (p < 0.001).

The correlation coefficient is 0.999 (P < 0.001) with well congruence by comparing the divergence time of species from the local clock estimation with the divergence time inferred from the fossil and molecular data (Figure 3B), indicating that the evolution of MCF keeps pace with metazoan evolution. The metabolic evolution of metazoans may be embodied in the functional evolution of MCF.

### The significant rise of hydrophobic residues at helix-helix interfaces

**Figure 4:**
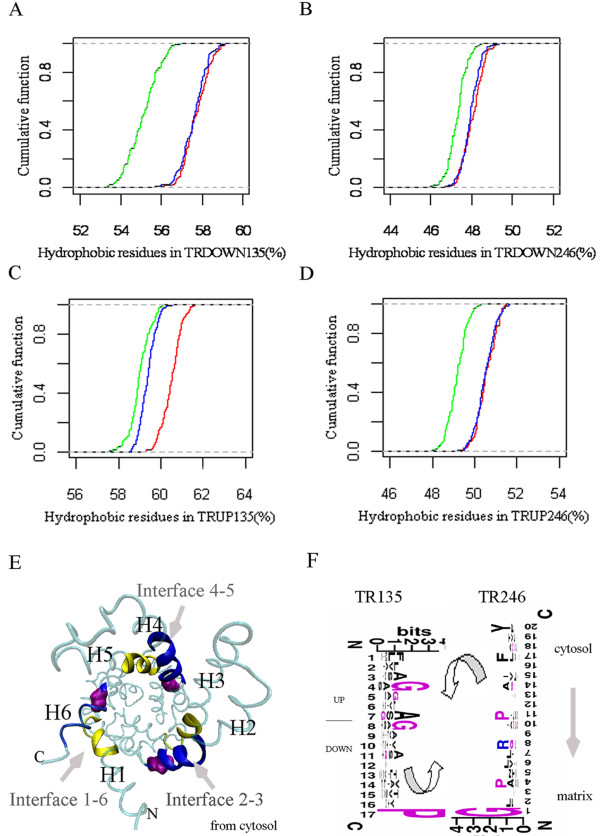
**Analysis of the AAC (amino acid component) in the transmembrane region of MCF**. (*A*) The cumulative distribution estimated from the proportion of the hydrophobic residues in TR_DOWN135_. (*B*) The cumulative distribution estimated from the proportion of the hydrophobic residues in TR_DOWN246_. *(C) *The cumulative distribution estimated from the proportion of the hydrophobic residues in TR_UP135_. *(D) *The cumulative distribution estimated from the proportion of the hydrophobic residues in TR_UP246_. Red represents Ver-endo; Blue represents Ver-eco; Green represents Non-ver. (E) The spatial distribution of transmembrane helices in the ADP/ATP carrier. Yellow represents TR_DOWN135 _(TR_DOWN1_, TR_DOWN3 _and TR_DOWN5_). Blue represents TR_UP246 _(TR_UP2_, TR_UP4 _and TR_UP6_). Purple represents P and G. Interface 1-6 consists of TR_DOWN1 _and TR_UP6_. Interface 2-3 consists of TR_UP2 _and TR_DOWN3_. Interface 4-5 consists of TR_UP4 _and TR_DOWN5_. (F) The logo chart of sequences in TR from metazoan.

The evolution of MCF was divided into three consecutive phases (see Methods) based on the fact that the evolution of MCF occurs in parallel to metazoan evolution. Statistics on the components of amino acids in different structural regions of MCF showed that the hydrophobic residues occupying a large proportion of TR had a significant rise from the invertebrate phase to the vertebrate phase (P < 0.001; Additional file 4A). There was a significant rise in the hydrophobic residues in TR_DOWN _(P < 0.001; Additional file 4B) too, while no significant changes in TR_UP _was observed. Thus, the region with a significant rise in hydrophobic residues was focused on TR_DOWN135 _(P < 0.001; Additional file 4C). There was no significant change in the proportion of charged residues and turn residues in TR. According to the method of deselecting species, three sequence pools were applied into the analysis of AACs. Non-ver was clearly separated from Ver-eco and Ver-endo according to the cumulative distribution of the proportion of the hydrophobic residues in TR_DOWN135 _(Figure 4A), while TR_DOWN246 _did not show a similar variation (Figure 4B). This result further demonstrated that the number of hydrophobic residues was significantly increased in TR_DOWN135 _during vertebrate evolution.

Interestingly, TR_UP246 _showed a similar distribution (Figure 4D) with TR_DOWN135_, indicating that the number of hydrophobic residues was increased in TR_UP246 _during vertebrate evolution too. Although TR_DOWN135 _and TR_UP246 _faced opposite directions (Additional file 1 and Figure 4E), the spatial distributions in the structure of ADP/ATP carrier clearly showed that they were close to each other and formed three helix-helix interfaces (Figure 4E). The conserved P and G (Figure 4F) in TR_246 _could serve as the hinges between TR_UP246 _and TR_DOWN246 _(Figure 4E) and made the helices in TR_UP246 _tilt to the TR_DOWN135 _sides (Figure 4E). TR_DOWN246 _participated in the formation of helix-helix interfaces with TR_DOWN135 _as well as TR_UP246 _(Additional file 5A). However, TR_UP135 _had no similar properties according to its space distribution (Additional file 5B). So, TR_DOWN135 _served as links between TR_UP246 _and TR_DOWN246 _and played a vital role in the formation of helix-helix interfaces, indicating the potential functional significance of the increased hydrophobic residues in TR_DOWN135_.

### The "tight" structure of TR during vertebrate evolution

**Figure 5:**
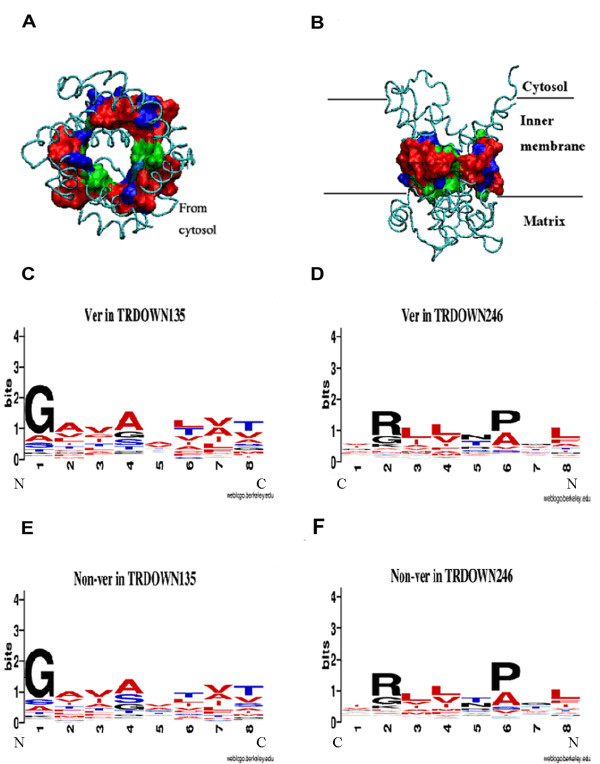
**The amino acid's spatial positioning and its evolutionary analysis in TR_DOWN_**. (*A*) *ADP/ATP *carrier viewed from the cytosol. The surf region represents TR_DOWN_. Red indicates hydrophobic residues. Blue indicates hydrophilic residues. Green indicates other residues. (*B*) The *ADP/ATP *carrier viewed from the matrix. Geno3D was used in the homology modelling of protein [61]. All the protein structures including the following structure were drawn by VMD [62]. (*C*) The logo chart of sequences in TR_DOWN135 _from vertebrates. (*D*) The logo chart of sequences in TR_DOWN246 _from vertebrates. (*E*) The logo chart of sequences in TR_DOWN135 _from invertebrates. (*F*) The logo chart of sequences in TR_DOWN246 _from invertebrates. Red indicates hydrophobic residues, blue indicates hydrophilic residues, and black indicates other residues. The logo chart describes the residue changes in the same vertical plane according to the space structure orientation of TR_DOWN _for metazoan evolution. WebLogo was applied into the logo analysis [21]. Ver indicates vertebrates, while Non-ver indicates invertebrates.

According to the orientation of the three dimensional structure of the *ADP/ATP *carrier, TR_DOWN _mainly consists of hydrophobic residues and some hydrophilic residues (Figure 5A and 5B). The changes of the type and number of hydrophobic residues in TR_DOWN _affected the connection among α-helices and further determined direct the degree of compactness of TR_DOWN _since hydrophobic residues in TR_DOWN _accounted for the connection among α-helices (Figure 5A and 5B). These hydrophobic residues were distributed in approximately the vertical planes along the central axis from the cytosol to the matrix (Figure 5B).

Furthermore, the logo [21] analysis based on all the sequences in the TR_DOWN _of MCF showed not only the enrichment of the hydrophobic residues at the same site or contiguous site in TR_DOWN_, but also the transformation of hydrophilic residues to hydrophobic residues in TR_DOWN135 _at sites 2, 3, 4, 6 and 7 during metazoan evolution (Figure 5C and 5E). The hydrophobic effect was lost once the helices were inserted into hydrophobic bilayers as a driving force for the helix association of integral membrane proteins [22] such as the mitochondrial inner membrane protein, MCF. The helix association then occurs through a combination of hydrogen-bonding, electrostatic, and van der Waals interactions [22]. Due to the enrichment of the hydrophobic residues in position 3, 4, 6, 8 of TR_DOWN246 _(Figure 5D and 5F) and the enrichment of the hydrophobic residues in position 4, 5, 7, 8 of TR_UP246 _(Additional file 6), we postulated that the methyl groups of the side chain of the hydrophobic residues might interact with the methyl groups of the hydrophobic residues from the neighboring residues in TR_DOWN135 _by van der Waals interactions to maintain the helix-helix interactions. The experimental evidence showed that A134 of bovine mitochondrial oxoglutarate carrier potentially engaged in inter-helical interactions as well as other key residues in the odd-numbered transmembrane α-helices [10]. Further, we found that A134 was located rightly at the TR_DOWN135 _and evolved from S at the same site of invertebrate (Additional file 7), which indirectly supported our hypothesis that the increase of hydrophobic residues would bring in more methyl groups, further strengthening the accumulated effect of the van der Waals interaction among them and generating a more "tight" TR structure.

According to the calculation of packing values of the residues in membrane proteins [22], TR_DOWN135 _in vertebrates had a significantly higher packing value (0.396) than that of invertebrates (0.383). This result thus further indicates that vertebrates have a more "tight" TR structure of MCF than invertebrates.

### Enhancement of substrate selectivity

**Figure 6:**
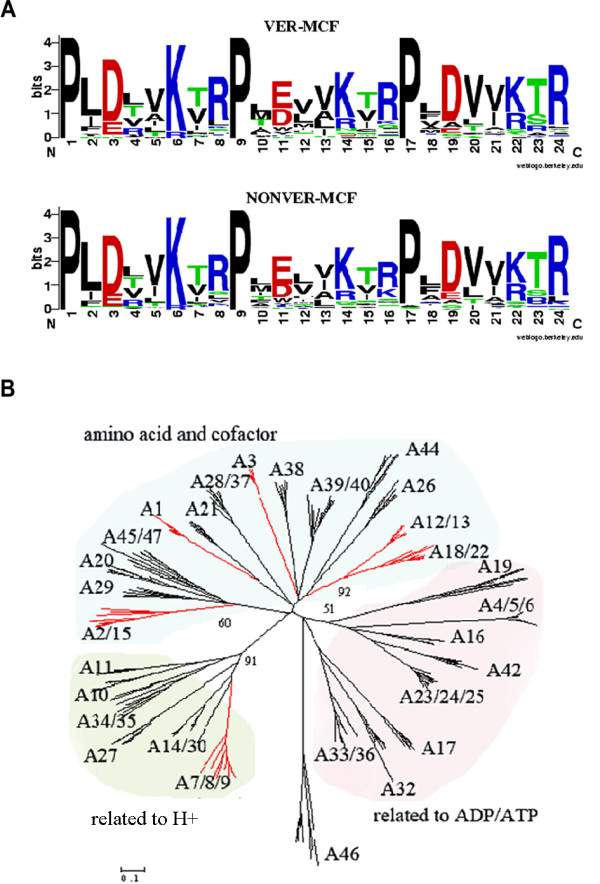
**The analysis of substrate transport of MCF**. (*A*) The logo chart of three tandem concatenated motifs of MCF for metazoan evolution. Three motif sequences constituting the conical pit were united end to end to form a mark sequence to research the variation in the substrate transported by MCF. WebLogo [21] was applied into the logo analysis. VER-MCF indicates a mark sequence of MCF in vertebrates. NONVER-MCF indicates a mark sequence of MCF in invertebrates. (*B*) Evolutionary pattern of substrate transport of MCs. A phylogenetic tree was constructed using MEGA4.0 [26] (Bootstrap = 1000, Poisson correction) based on TR sequences from 44 carriers in SLC25 and their orthologous sequences in nine species (44 Χ 9). A represents a subfamily. The index in SLC25 (the name of MCF in human) was used to denote the subfamily, such as A7, 8, 9 (representing the orthologous sequences of *UCP*1, 2, 3 in human, respectively). A47 represents the subfamily of Hepatocellular carcinoma-down-regulated mitochondrial carrier protein in human. The red branch indicates the carriers related to H^+^. Bootstrap values greater than 50 for selected nodes with clusters including more than four subfamilies were indicated.

The salt network formed by the positive acids and negative acids (Additional file 8A) from three motif sequences (Px(D/E)xx(K/R)) constituting the conical pit of MCF (Additional file 8C) is important for transporting substrates [23-25]. Thus, the tandem concatenated sequences from three motifs were selected to serve as the indicators selecting the substrates. The Logo analysis on the mark sequences of MCF as a whole in metazoans showed a high similarity (Figure 6A), indicating that the types of substrates transported by MCF are not different. This is in agreement with the strong purifying selection (Additional file 3) occurring in the evolution of MCF in metazoan.

A phylogenetic tree was constructed using MEGA4.0 [26] based on TR sequences from 44 carriers in SLC25 and their orthologous sequences in nine species (44 Χ 9) (Figure 6B). As can be seen, its branches were labelled according to previous reports related to substrates transported by MCs [1,2]. The tree clearly showed three clusters that correspond to three categories of substrates, namely ADP/ATP, amino acid and cofactor, and H^+^. According to the E-value of each carrier obtained by BLAST in all the fungal genomes database, we calculated the average E-values of representative carriers for each cluster, showing that the ADP/ATP cluster was 10^-50.56, ^the amino acid and cofactor cluster was 10^-43^, and the H^+ ^cluster was 10^-38.35^. The results showed that the ADP/ATP cluster was an older cluster, while the H^+ ^cluster was relatively younger cluster. Combined with the size of substrates transported by MCs [1], we found that the size of substrates transported by members of MCs continuously diminished throughout the evolution (Figure 6B).

Proline served as the hinge between TR_DOWN135 _[23] and the label sequences of Px(D/E)xx(K/R) [24,27] (Additional file 8). The conserved P and G (Figure 4F) in TR_246 _(Figure 4E and Figure 4F) could also serve as the hinges between TR_UP246 _and TR_DOWN246_. Furthermore, these conserved P and G in the transmembrane α-helices (Figure 4F) constituted the P-G level 1 located at approximately the midpoint of transmembrane region and the P-G level 2 located at approximately the bottom of transmembrane region [28]. The two P-G levels [28] made the TR_DOWN _relatively independent and less affected by the structural changes due to helix tilt during the substrate transport process. So, the increased hydrophobic residues at the specific helix-helix interfaces could enhance the compactness degree in TR_DOWN _and strengthened the interaction between substrates and substrates contacting sites [4] of the MCF, thus allowing the passing of the substrates with the given size, in agreement with the trend of continuously diminishing size of substrates (Figure 6B).

## Discussion

### MCF acts as the molecular fossil of metazoan

The rise of oxygen concentration appears to have precipitated a massive increase in metabolic complexity, culminating in the rise of metazoans around 600 million years ago [29]. Feedback in the rise of oxygen concentrations has led to increasing metabolic complexity [29] and shows the existence of the intrinsic molecular mechanism of continuously adapting to the rise of oxygen concentration. We found that many gene duplication events of MCF occurred during the early period of vertebrate evolution, especially during the Ediacarian and Cambrian periods (Additional file 2). Further, the molecular evolution of MCF keeps pace with macroevolution (Figure 3). MCF located in mitochondria has a direct effect on metabolic activity [1,2], and thus this feedback mechanism may be embodied in the functional evolution of MCF. These data make the MCF act as the molecular fossil for exploring metabolic complexity in metazoan.

### Enhancement of substrates selectivity correlates to the rise of oxygen concentration

The enhancement of the substrate selectivity of MCF could partially explain the feedback mechanism evolved in metabolic evolution from the viewpoint of providing energy and reducing damage. From the view of providing energy, if mitochondria can be regarded as an energy-producing factory, its core department is the electron-transport chain. The enhancement of substrate selectivity of MCF allowed for more frequent communication between the mitochondrial matrix and the IMM. In addition, this increase of substrate selectivity provided more energy materials to the energy-producing factory by promoting the operation efficiency of this transport machine. Thus, the increase in the concentration of oxygen as the last electron receiver in the electron-transport chain [30,31] would produce more abundant energy to satisfy physiological needs.

Oxygen participates in the provision of energy for the evolution of life but also brings by-products, such as superoxide and peroxides [29]. The enhancement of substrate selectivity of MCF offers a possible way to reduce oxygen damage. It is the tendency towards strengthening of the substrate selectivity of MCF that makes the enhancement of selectivity of the H^+ ^substrate. Uncoupling protein is generally considered as responsible for H^+ ^conductance [32,33]. No specific uncoupling protein is responsible for the proton conductance of yeast mitochondrion [34]. The recent research represented that lamprey, generally considered as the most primitive vertebrate, had the uncoupling protein (UCP) transporting H^+ ^[35], at least indicating the enhancement of selectivity of the H^+ ^substrate. We also found the UCP subfamily was diverged approximately 637 million years ago (Additional file 2), in good agreement with around 600 million years ago when the rise of oxygen concentration culminates in the rise of metazoans. This good correlation further indicate that the rise of oxygen concentration may induces the enhancement of selectivity of the H^+ ^substrate, which reduces oxygen damage by affecting the IMM potential, such as the role of the UCP located in the H^+ ^cluster in reducing superoxide production and production of ROS [36-38].

In view of the important role of MCF as a molecular fossil in metabolism, we postulate that oxygen could be an environmental factor affecting the variation of substrate transport of MCF. Our studies explore the functional mechanism of oxygen on the evolution of MCF from the viewpoint of the enhancement of substrate selectivity of MCF.

## Conclusions

The more "tight" TR structure generated by the increase of the hydrophobic amino acids at specific helix-helix interfaces enhances the substrate selectivity of MCF, which reflects the evolutionary trajectory of MCF. Because the goal of this study was to understand the variation in the structure of MCF from the level of sequence, our findings might provide the more specific mutation sites for laboratory experiments to improve experimental efficiency.

## Methods

### Partitioning evolutional processes and obtaining orthologous sequences

The orthologous sequences of human MCF (SLC25) were obtained from Swiss-Prot/TrEMBL and GenBank using the BLAST network service [39,40]. The complexity of metabolism was reflected by the evolutional process of metazoan, and thus the orthologous sequences from metazoan were studied exclusively. In view of the facts that sequence similarity is close to 30% between members of SLC25 [41], the shorter process of metazoan evolution has a lower sequence variation and the orthologous gene itself has the a higher sequence similarity, the E-value of BLAST was set to 10^-50 ^to exclude the interference from many nonhomologous genes. For cases when there was more than one sequence with a high similarity to human members, the sequence with the highest similarity was selected as the orthologous gene.

The emergences of vertebrates and endotherms indicate the two great leaps in metazoan evolution, and thus metazoan evolution was divided into three consecutive phases named the Non-ver (invertebrate) phase, the Ver-eco (vertebrate ectotherm) phase and the Ver-endo (vertebrate endotherm) phase. As current databases do not provide sequence data of MCF for more than three ectotherm species, we only chose three fully annotated species from three partitioned phases to meet the statistical requirements of ANOVA. *Caenorhabditis elegans, Drosophila melanogaster *and *Strongylocentrotus purpuratus *were used to represent the Non-ver phase. *Tetraodon nigroviridis, Danio rerio *and *Xenopus tropicalis *were used to represent the Ver-eco phase. *Bos Taurus, Mus musculus*, and *Homo sapiens *were used to represent the Ver-endo phase. The number of carriers from the nine species are as follows: human (44), mouse (44), bovine (40), frog (43), zebrafish (44), fugu (41), sea urchin (22), fly (26), and worm (25). A list of sequence accessions could be found in Additional file 9.

### Classification of the whole structural region

In accordance with the generally accepted secondary structure of MCF [1,2,13,23,27,41], we defined amino-ends, carboxyl-ends and two hydrophilic regions (n, c, loop2, loop4) facing the cytosol space as NCLOOP24; transmembrane regions consisting of six α-helices as TR; and three hydrophilic regions facing matrix space as LOOP135 (loop1, loop3, loop5) (Additional file 1). Six transmembrane α-helices were defined according to the classification of TR [42] and the c-terminal definition of three odd α-helices [41]. The example of TR classification could be found in the CLUSTAL alignment among the oxoglutarate/malate sequences in metazoan species (Additional file 7). TR_135 _represents three odd α-helices. TR_246 _represents three even α-helices. TR was separated into two parts along the vertical direction from cytosol to matrix (Additional file 1). TR_UP _indicates the upper part facing cytosol. TR_UP _was further divided into two parts: TR_UP135 _was made up of three odd α-helices, while TR_UP246 _consisted of three even α-helices. TR_DOWN _represents the lower part of TR which faces the mitochondria matrix. TR_DOWN _was further divided into two parts: TR_DOWN135 _was made up of three odd α-helices, while TR_DOWN246 _consisted of three even α-helices.

### Gene duplication and natural selection detection

Gene duplication events were detected through the construction of phylogenetic trees of gene families [15]. The acceptance criterion was that the orthologous subfamily emerging from a duplication event had at least two species. The topology of the phylogenetic tree of the candidate family was constructed by the neighbor-joining method [43] (bootstrap = 500, JTT matrix).

The nonsynonymous substitution (those causing amino acid-alterations) to synonymous substitution (silent) rate ratio (ω = *d_N_/d_S_*) provides a sensitive measure of natural selection at the protein level, with ω values of 1, > 1, and < 1, indicating neutral evolution, positive selection and purifying selection, respectively [44]. We used branch-site model A [44,45] to detect the type of natural selection of TR. This model has been reported to be more sensitive to the detection of positive selection than previous models including branch models [46] and site models [47], which may be useful in detecting positive selection after gene duplication in gene family evolution [44]. We used a branch-site test of positive selection [45] to construct a likelihood ratio test between Model A with ω_2_≧1(alternative model,) and Model A1 with ω_2 _= 1 fixed (null model). The test was done by comparing the difference of likelihood values 2λ to a χ2 distribution of 1 degree of freedom. The critical values of 2λ at the 5% and 1% level are 2.71 and 5.41, respectively [48]. Sites that could be under positive selection were identified with a Bayes Empirical Bayes approach [49]. In addition, synonymous (*d_S_*) and nonsynonymous (*d_N_*) substitution rates were estimated using the methods of Yang and Nielson [50] as implemented in yn00 using PAML software [48].

### Time scale and molecular clock estimation

The time scale estimation of gene duplication with multiple calibrations was implemented in the codeml program [51] of the PAML package (version 4) [48]. We adopted several calibrations inferred from both fossil data and molecular data [52,53], such as the primate-rodent (91 Myr ago), and vertebrate-fly (993 Myr ago) calibrations. The mtREV24 model [54] was applied as a model of amino acid substitution. We used both global clock and local clock methods to conduct the time scale estimation of gene duplication [55]. Linear regression was applied to test the congruence between global clock and local clock.

We applied the above-mentioned method to the molecular clock estimation to infer whether the evolution of MCF keeps pace with macroevolution. Nine species could be found in the small number of orthologous sequences of human MCF, barely enough on behalf of MCF. Therefore, six species (worm, fruitfly, zebrafish, frog, mouse, and human) were selected to narrow the range of species. Then 33 orthologous sequences were found for each species. Further, the tandem concatenated sequence, which consisted of 33 orthologous TR sequences in each species, was applied in the molecular clock estimation.

### Analysis of the feature of the sequence from the structure of MCF

The statistics of the amino acid component (AAC) were conducted according to the classification of the structure of MCF. Hydrophobic residues were based on the hydrophobicity table [56]. Our compiled program using perl 5.8.7 was applied to the AAC statistic with two approaches of selecting and de-selecting species, respectively. Firstly, the statistics with selecting species were based on the above-mentioned nine species. A one-way ANOVA, followed by the Holm-Sidak test for multiple comparisons, was conducted using SigmaStat Windows Version 3.5 to evaluate differences in mean values among groups. The downloaded orthologous sequences according to 10^-50 ^(not including sequence fragments and the sequences with obvious deletion in TR or conical pit region) were divided into the three sequences pools that were in the Non-ver pool (192), the Ver-eco pool (164), and the Ver-endo pool (208). A list of sequence accessions could be found in Additional file 10. The mean value of the percentage of the relative AAC was calculated from 132 (44 carriers × 3 species) sequences sampled randomly from three pools, respectively. This process was repeated for 132 times. The cumulative distribution function in the R language was applied to the analysis of the cumulative distribution of the mean values obtained.

Helix packing is important in the folding, stability, and association of membrane proteins [22,57]. The packing value correlates positively with the degree of compactness of TR [22,58]. Therefore, the quantitative statistics of packing values of the membrane protein can partially reflect the degree of compactness of TR. By studying the packing trait on the membrane protein helix from the obtained structure data of the membrane complex [22], it was able to obtain the packing values of the helix residues of TR. These data were applied to determine the average packing values of TR in MCF, indicating changes in the degree of compactness of TR during metazoan evolution.

### The evolution of substrate transport of MCs

A phylogenetic tree was constructed using MEGA4.0 [26] based on TR sequences from 44 carriers in SLC25 and their orthologous sequences in nine species (44 Χ 9). In view of the large number of sequences, the relatively low number of amino acids (the average length of the sequences used was 111 amino acids), and the relatively high divergence among the sequences, the neighbor-joining method was selected to generate the phylogenetic tree with the Poisson correction used to measure the pairwise sequence distances [59,60]. Other tree-generating algorithms (minimal evolution, UPMGA and maximum likelihood) resulted in phylogenetic trees with a similar topology regarding the main branches generated by the neighbor-joining method. According to previous reports about the function of the members of MCF [1,2], we could obtain the pattern of substrate transport of MCF.

As this family is exclusive to eukaryotes [4], we took all the fungal genomes in NCBI, the primitive species in eukaryotes, as a reference from which to estimate the ancient level of MCs. The sequences of SLC25 members were applied into the BLAST network service in all the fungal genomes in NCBI to obtain the corresponding list of E-values. The smallest 10 E-values on the list were used to determine the mean value statistics. The smaller the mean value, the higher the similarity between the sequences, and the older the carrier.

## Abbreviations

MCF: mitochondrial carrier family; MC: mitochondrial carrier; SLC25: solute carrier family 25; TR: transmembrane region; IMM: inner membrane of mitochondria; UCP: uncoupling protein; ADP: adenosine-5'-diphosphate; ATP: adenosine-5'-triphosphate; ROS: reactive oxygen species; AAC: amino acid component.

## Authors' contributions

MG and JL performed the analyses and drafted the manuscript. MW helped to draft the manuscript. MG, JW and KZ revised the manuscript. JW and KZ supervised the study. CZ initiated and supervised the project. All authors approved the final manuscript.

## Supplementary Material

Additional file 1 **Two-dimensional structure of MCF**. The structure of MCF consists of the three segments that are NCLOOP24 facing the space of cytosol, TR located in the inner membrane, and LOOP135 facing the space of matrix, respectively. TR is made up of six α-helices represented by six bars. Dark dot represents individual amino acid. Click here for file

Additional file 2 **Gene duplication events and the divergence time of MCs**. Orthologous TR sequences from 44 members of SLC25 were applied into the construction of the phylogenetic trees to identify gene duplications events. 15 gene duplication events were detected in 12 independent trees. Arrow indicates a gene duplication event with the divergence time (Myr ago). The index in SLC25 (the appellation of MCF in human) was used to denote the corresponding orthologous sequences, such as A7, 8, 9 (representing the orthologous sequences of UCP1, 2, 3 in human, respectively). A47 represents the subfamily of Hepatocellular carcinoma-down-regulated mitochondrial carrier protein in human. Click here for file

Additional file 3 **Likelihood scores, model parameters, and likelihood-ratio test results for branch-site model analyses**. A indicates the orthologous sequences of member of SLC25. The index in SLC25 (the appellation of MCF in human) was used to denote the corresponding orthologous sequences, such as A7, 8, 9 (representing the orthologous sequences of UCP1, 2, 3, respectively). We used a branch-site test of positive selection [45] to construct a likelihood ratio test between Model A with ω_2_≧1(alternative model,) and Model A1 with ω_2 _= 1 fixed (null model). The test was done by comparing the difference of likelihood values 2λ to a χ2 distribution of 1 degree of freedom. The critical values of 2λ at the 5% and 1% level are 2.71 and 5.41, respectively [48]. Click here for file

Additional file 4 **Analysis of the AAC (amino acid component) in MCF**. (*A*) The mean percentage of the hydrophobic residues in TR. (*B*) The comparison of the mean percentage of the hydrophobic residues in TR_UP _and TR_DOWN_. (*C*) The comparison of the mean percentage of the hydrophobic residues in TR_DOWN135 _and TR_DOWN246_. The groups were evaluated using a one way ANOVA followed by the Holm-Sidak test for multiple comparisons. n = 132. Bars represent the mean ± s.e.m, while an asterisk indicates P < 0.001 in comparison to the Non-ver phase. Click here for file

Additional file 5 **The spatial distribution of transmembrane α-helices in the human ADP/ATP carrier**. *(A*) The human ADP/ATP carrier viewed from the lateral side. Yellow represents TR_DOWN135_. Blue represents TR_246. _*(B*) The human ADP/ATP carrier viewed from the cytosol. Yellow represents TR_UP135._ Click here for file

Additional file 6 **The logo chart of sequences in TR_UP246_**. *(A) *The logo chart of sequences in TR_UP246 _from Ver. *(B) *The logo chart of sequences in TR_UP246 _from Non-ver. Red indicates hydrophobic residues, blue indicates hydrophilic residues, and black indicates other residues. The logo chart describes the residue changes in the same vertical plane according to the space structure orientation of TR_UP246 _for metazoan evolution. WebLogo [21] was applied into the Logo analysis. Ver indicates vertebrates, while Non-ver indicates invertebrates. Click here for file

Additional file 7 **The CLUSTAL alignment among the oxoglutarate/malate sequences in metazoan species**. The sequences with fragment and obvious deletion in TR and conical pit region (CPR) were not included. CLUSTAL W [63] was applied to multiple sequence alignment. The marks "*", ".", and ":" stand for the identical amino acids, relative, and similar amino acids. CPR consists of three motif sequences (Px(D/E)xx(K/R)) that are located at the C-terminal of TR1, TR3 and TR5, respectively. The sequence accessions according to the species order above were as followed: Q02978, Q9CR62, P97700, NP_777096.1, NP_001090497.1, AAH71521.1, CAF90256.1, XP_002610854.1, XP_001639936.1, XP_001867726.1, NP_651703.1, NP_493694.2, XP_002571870.1 and XP_001893008.1. The detailed classifications of TR sequences and CPR in the oxoglutarate/malate carrier are applicable to other MCs too. Click here for file

Additional file 8 **The structures of the CPR in the ADP/ATP carrier**. (*A*) The ADP/ATP carrier in humans viewed from the lateral side. The surf region represents the CPR. Red indicates negative acids, blue indicates positive acids, white indicates non-polar residues, and green indicates polar residues. (*B*) The ADP/ATP carrier in human viewed from the cytosol. (*C*) TR_DOWN _and CPR viewed from the cytosol. Proline serves as the hinge between TR_DOWN _and CPR. (*D*) TR_DOWN _and CPR viewed from the matrix. Click here for file

Additional file 9 **The accessions list from the selecting species**. The details about selecting species are available in the method section. Click here for file

Additional file 10 **The accessions list from the deselecting species**. The details about deselecting species are available in the method section. Click here for file
