# Supplementary figures and images for: The evolutionary trajectory of mitochondrial carrier family during metazoan evolution
Source: BMC Evol Biol. 2010 Sep 16;10:282. doi: 10.1186/1471-2148-10-282 (PMC2949871; doi:10.1186/1471-2148-10-282)

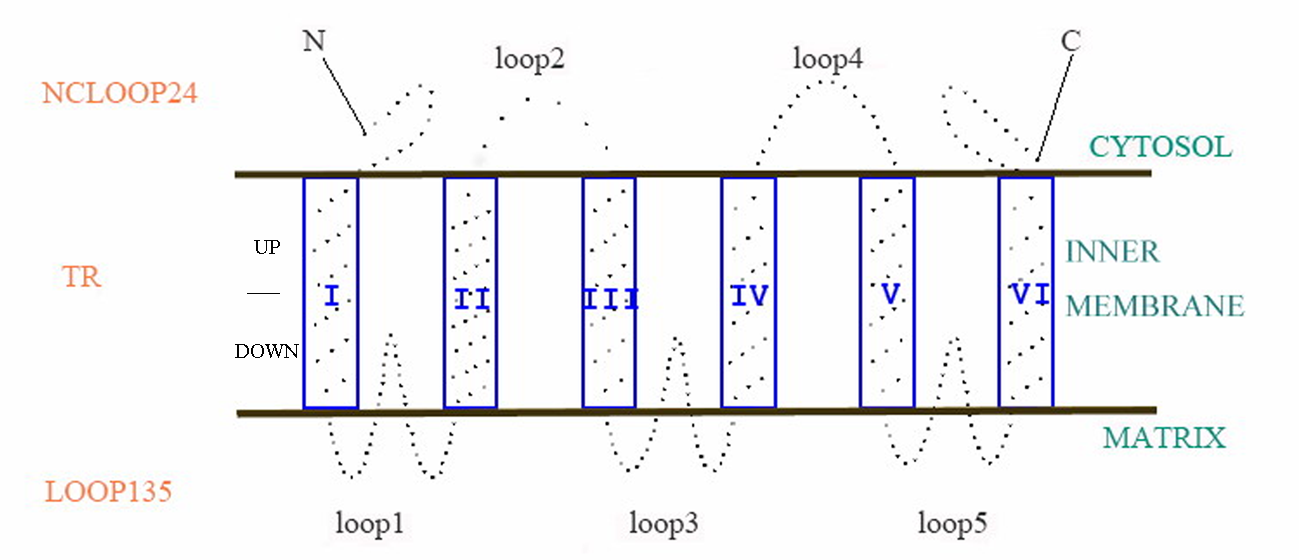

Supplement: Additional file 1 — Two-dimensional structure of MCF. The structure of MCF consists of the three segments that are NCLOOP24 facing the space of cytosol, TR located in the inner membrane, and LOOP135 facing the space of matrix, respectively. TR is made up of six α-helices represented by six bars. Dark dot represents individual amino acid. [file 1471-2148-10-282-S1.TIFF]

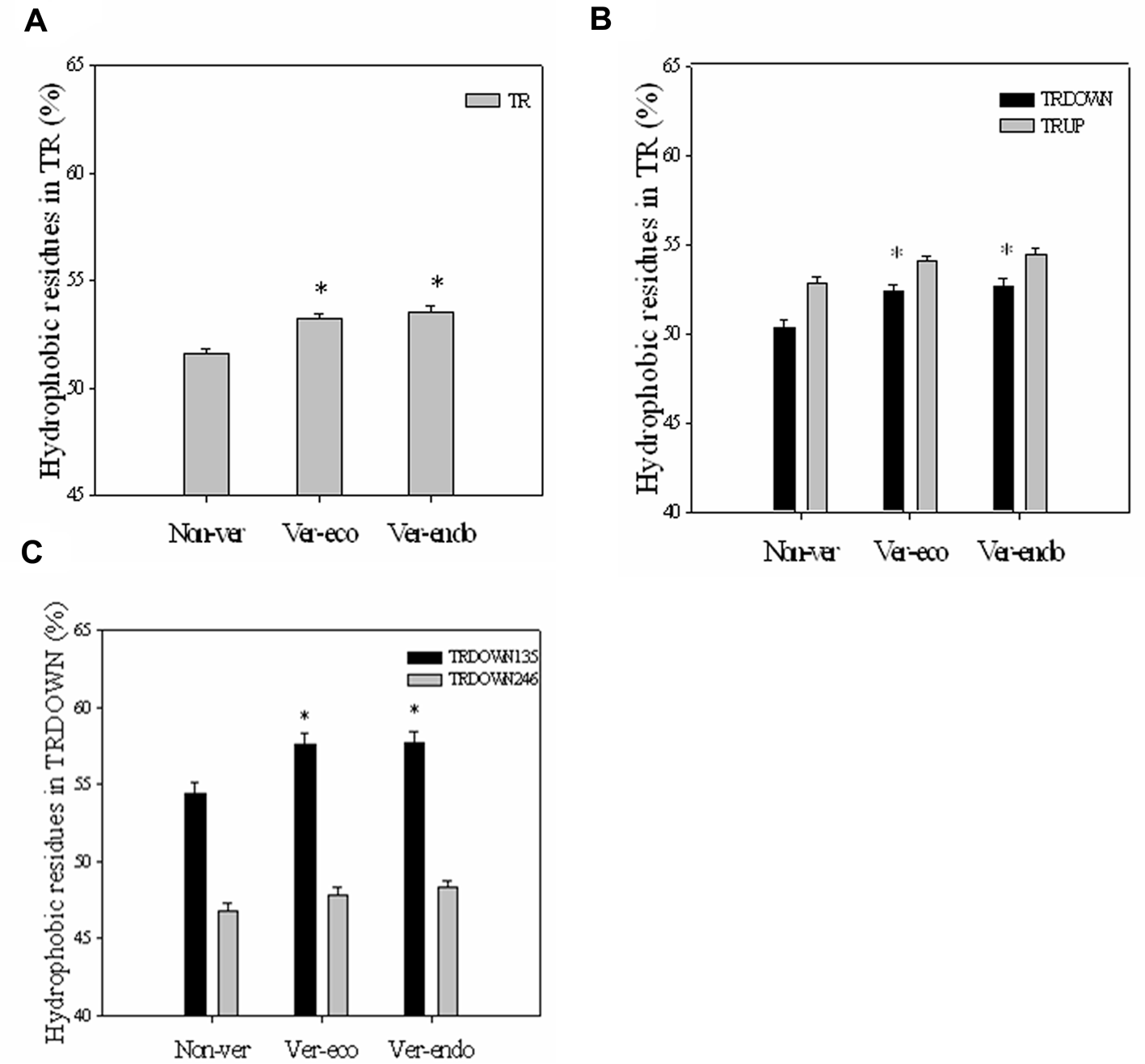

Supplement: Additional file 4 — Analysis of the AAC (amino acid component) in MCF. (A) The mean percentage of the hydrophobic residues in TR. (B) The comparison of the mean percentage of the hydrophobic residues in TRUP and TRDOWN. (C) The comparison of the mean percentage of the hydrophobic residues in TRDOWN135 and TRDOWN246. The groups were evaluated using a one way ANOVA followed by the Holm-Sidak test for multiple comparisons. n = 132. Bars represent the mean ± s.e.m, while an asterisk indicates P < 0.001 in comparison to the Non-ver phase. [file 1471-2148-10-282-S4.TIFF]

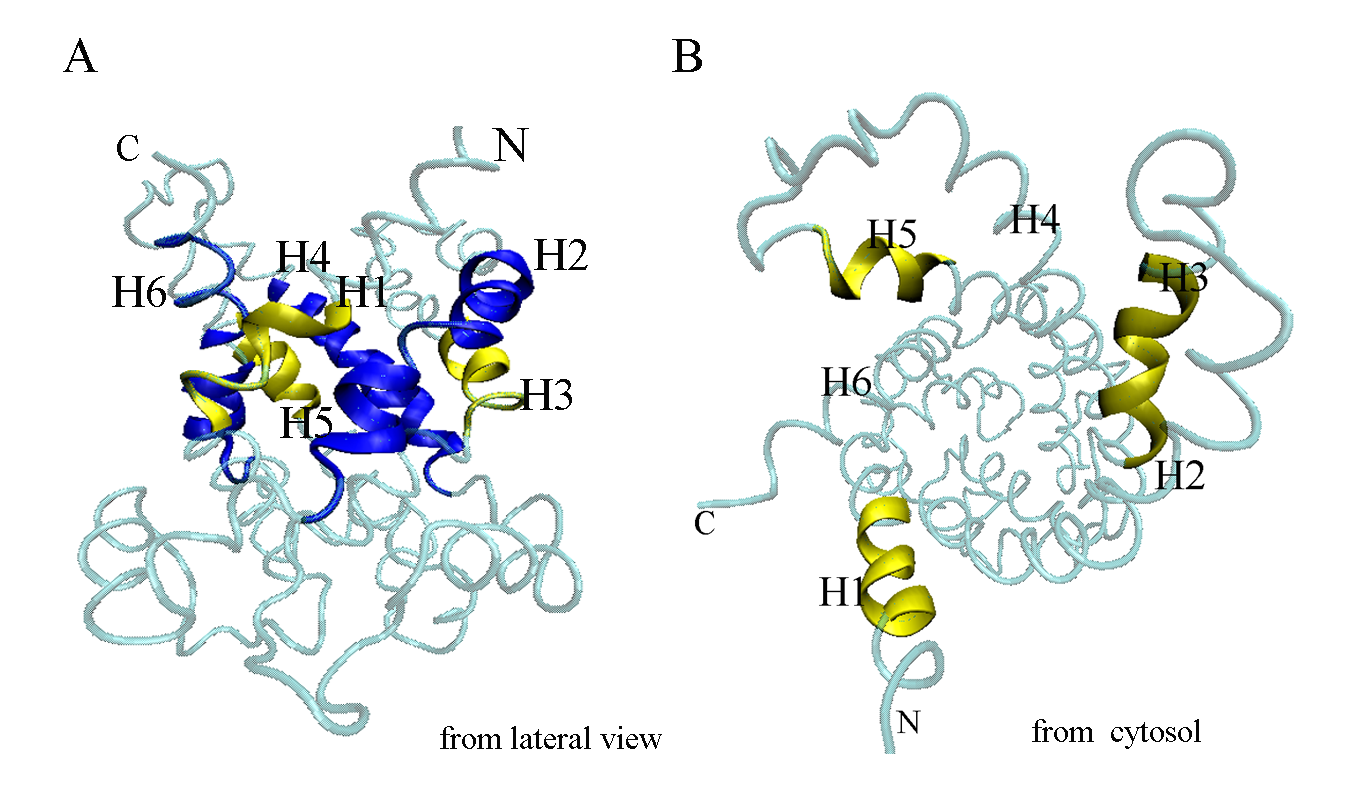

Supplement: Additional file 5 — The spatial distribution of transmembrane α-helices in the human ADP/ATP carrier. (A) The human ADP/ATP carrier viewed from the lateral side. Yellow represents TRDOWN135. Blue represents TR246. (B) The human ADP/ATP carrier viewed from the cytosol. Yellow represents TRUP135. [file 1471-2148-10-282-S5.TIFF]

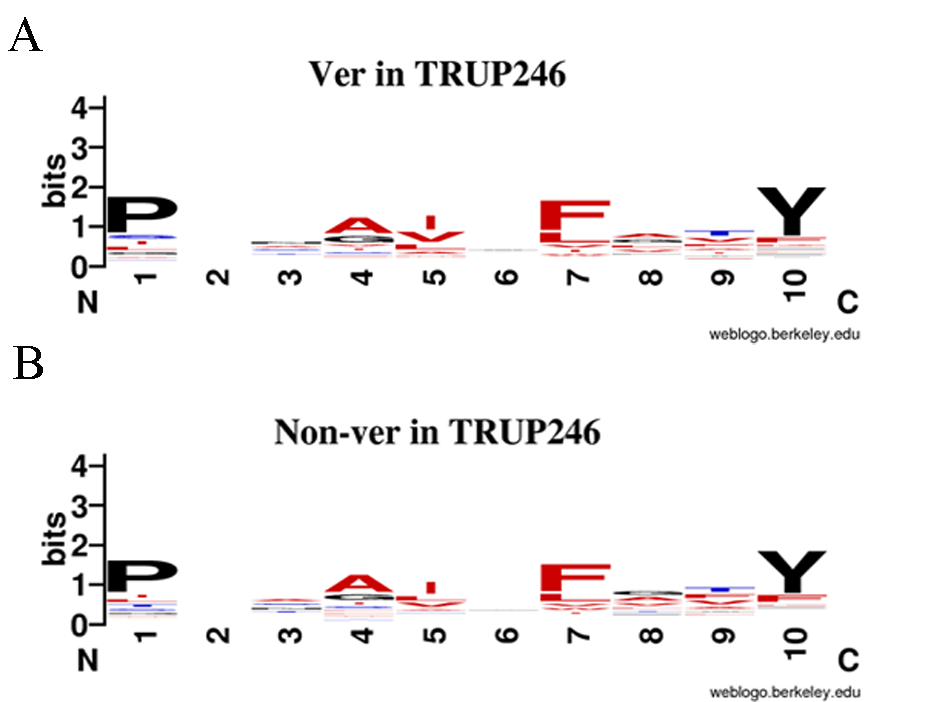

Supplement: Additional file 6 — The logo chart of sequences in TRUP246. (A) The logo chart of sequences in TRUP246 from Ver. (B) The logo chart of sequences in TRUP246 from Non-ver. Red indicates hydrophobic residues, blue indicates hydrophilic residues, and black indicates other residues. The logo chart describes the residue changes in the same vertical plane according to the space structure orientation of TRUP246 for metazoan evolution. WebLogo [21] was applied into the Logo analysis. Ver indicates vertebrates, while Non-ver indicates invertebrates. [file 1471-2148-10-282-S6.TIFF]

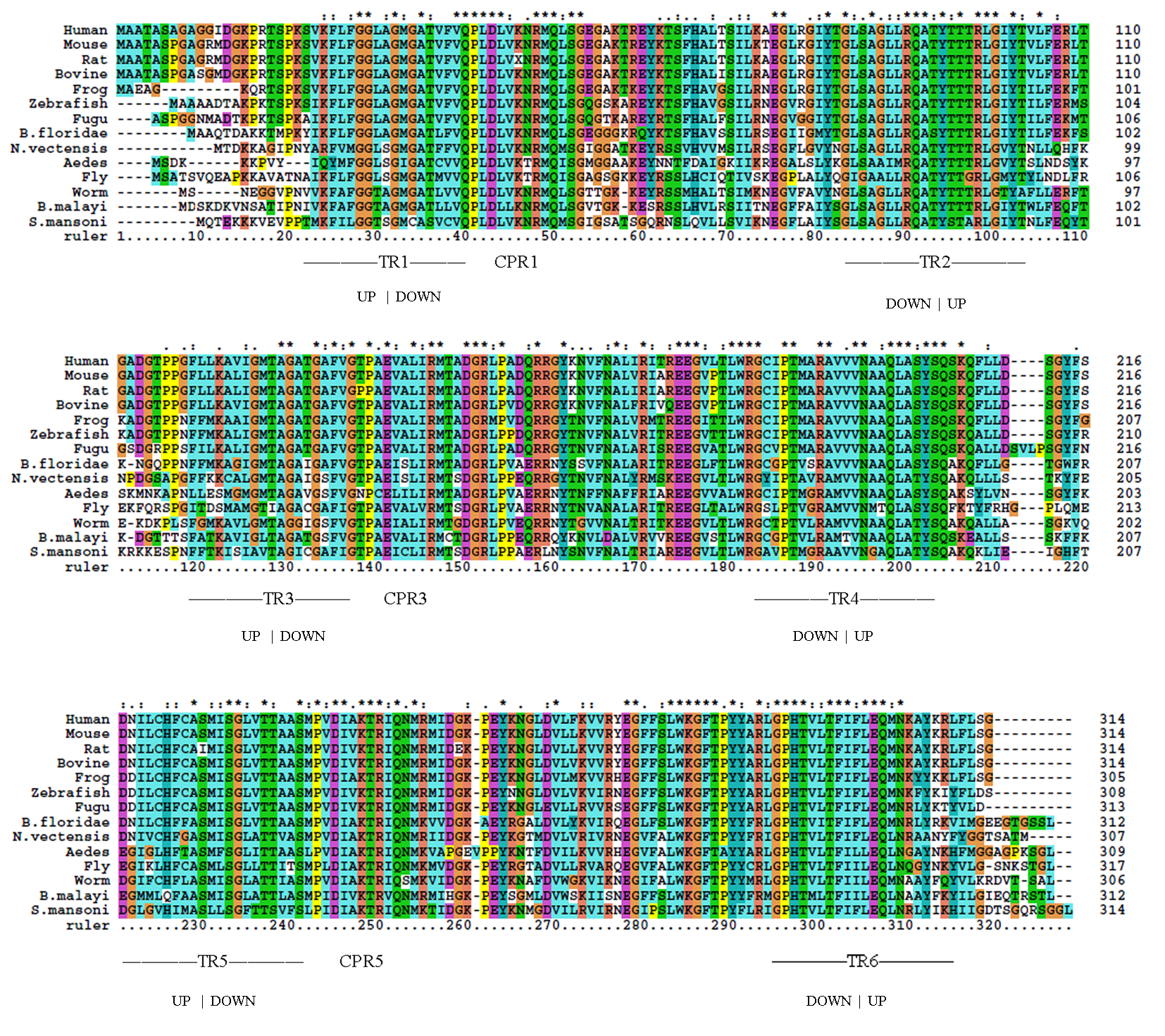

Supplement: Additional file 7 — The CLUSTAL alignment among the oxoglutarate/malate sequences in metazoan species. The sequences with fragment and obvious deletion in TR and conical pit region (CPR) were not included. CLUSTAL W [63] was applied to multiple sequence alignment. The marks "*", ".", and ":" stand for the identical amino acids, relative, and similar amino acids. CPR consists of three motif sequences (Px(D/E)xx(K/R)) that are located at the C-terminal of TR1, TR3 and TR5, respectively. The sequence accessions according to the species order above were as followed: Q02978, Q9CR62, P97700, NP_777096.1, NP_001090497.1, AAH71521.1, CAF90256.1, XP_002610854.1, XP_001639936.1, XP_001867726.1, NP_651703.1, NP_493694.2, XP_002571870.1 and XP_001893008.1. The detailed classifications of TR sequences and CPR in the oxoglutarate/malate carrier are applicable to other MCs too. [file 1471-2148-10-282-S7.TIFF]

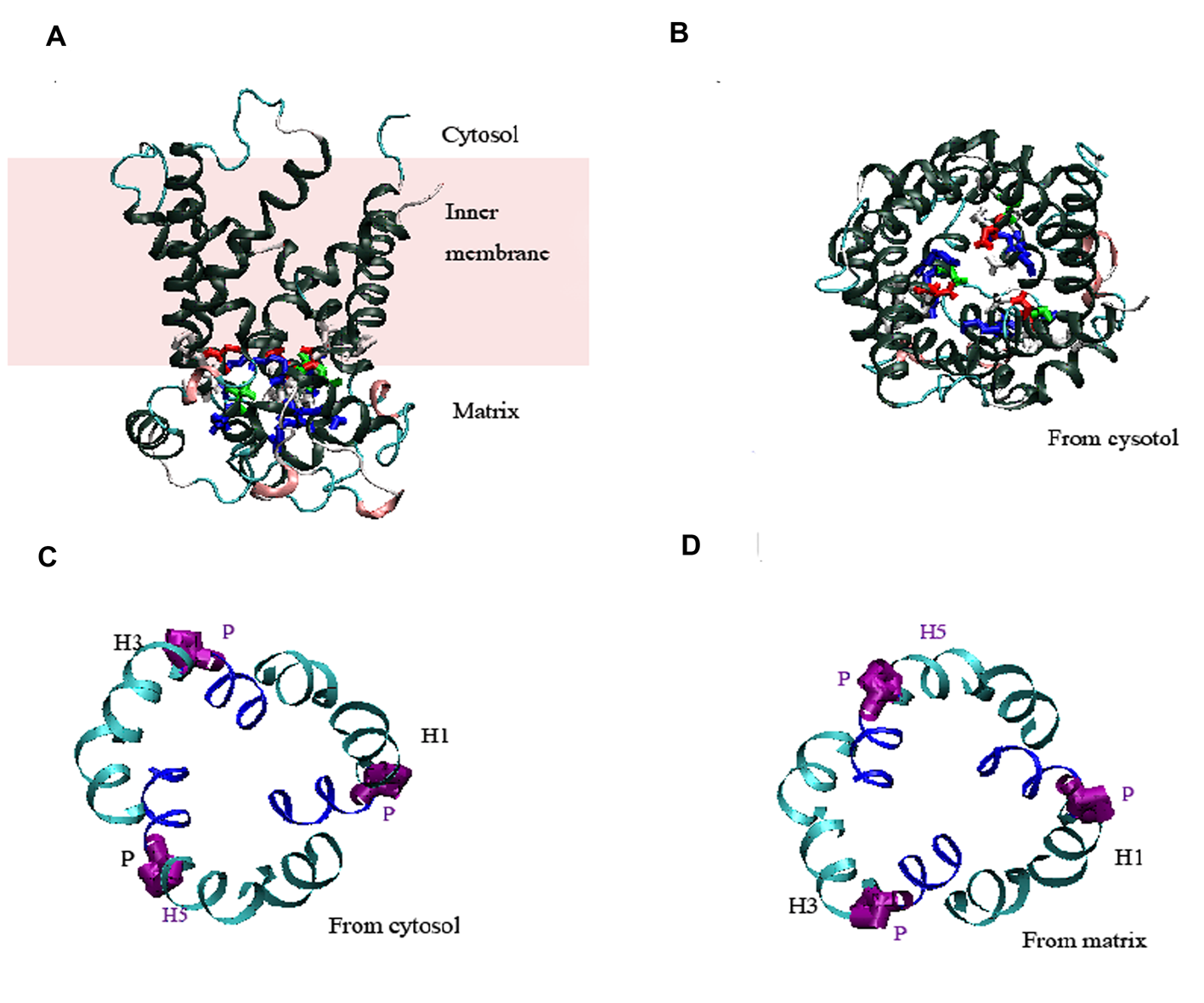

Supplement: Additional file 8 — The structures of the CPR in the ADP/ATP carrier. (A) The ADP/ATP carrier in humans viewed from the lateral side. The surf region represents the CPR. Red indicates negative acids, blue indicates positive acids, white indicates non-polar residues, and green indicates polar residues. (B) The ADP/ATP carrier in human viewed from the cytosol. (C) TRDOWN and CPR viewed from the cytosol. Proline serves as the hinge between TRDOWN and CPR. (D) TRDOWN and CPR viewed from the matrix. [file 1471-2148-10-282-S8.TIFF]
