# Supplementary material for: The evolutionary trajectory of mitochondrial carrier family during metazoan evolution
Source: BMC Evol Biol. 2010 Sep 16;10:282. doi: 10.1186/1471-2148-10-282 (PMC2949871; doi:10.1186/1471-2148-10-282)
Supplement: Additional file 2 — Gene duplication events and the divergence time of MCs. Orthologous TR sequences from 44 members of SLC25 were applied into the construction of the phylogenetic trees to identify gene duplications events. 15 gene duplication events were detected in 12 independent trees. Arrow indicates a gene duplication event with the divergence time (Myr ago). The index in SLC25 (the appellation of MCF in human) was used to denote the corresponding orthologous sequences, such as A7, 8, 9 (representing the orthologous sequences of UCP1, 2, 3 in human, respectively). A47 represents the subfamily of Hepatocellular carcinoma-down-regulated mitochondrial carrier protein in human. [file 1471-2148-10-282-S2.DOC]

1074

849

671

A23/24/25

A2/15

551

6474

637

551

A4/5/6

A7/8/9

455

677

A12/13

A28/37

359

872

A18/22

A14/30

756

615

A33/36

A34/35

401

534

A39/40

A45/47

**Additional file 2**

**Gene duplication events and the divergence time of MCs.** Orthologous TR sequences from 44 members of SLC25 were applied into the construction of the phylogenetic trees to identify gene duplications events. 15 gene duplication events were detected in 12 independent trees. Arrow indicates a gene duplication event with the divergence time (Myr ago). The index in SLC25 (the appellation of MCF in human) was used to denote the corresponding orthologous sequences, such as A7, 8, 9 (representing the orthologous sequences of UCP1, 2, 3 in human, respectively). A47 represents the subfamily of Hepatocellular carcinoma-down-regulated mitochondrial carrier protein in human.
