# Supplementary material for: The evolutionary trajectory of mitochondrial carrier family during metazoan evolution
Source: BMC Evol Biol. 2010 Sep 16;10:282. doi: 10.1186/1471-2148-10-282 (PMC2949871; doi:10.1186/1471-2148-10-282)
Supplement: Additional file 3 — Likelihood scores, model parameters, and likelihood-ratio test results for branch-site model analyses. A indicates the orthologous sequences of member of SLC25. The index in SLC25 (the appellation of MCF in human) was used to denote the corresponding orthologous sequences, such as A7, 8, 9 (representing the orthologous sequences of UCP1, 2, 3, respectively). We used a branch-site test of positive selection [45] to construct a likelihood ratio test between Model A with ω2≧1(alternative model,) and Model A1 with ω2 = 1 fixed (null model). The test was done by comparing the difference of likelihood values 2λ to a χ2 distribution of 1 degree of freedom. The critical values of 2λ at the 5% and 1% level are 2.71 and 5.41, respectively [48]. [file 1471-2148-10-282-S3.DOC]

| **Branch-site model A** | **Model parameters** | | | | | **Likelihood ratio test details** | | |
| --- | --- | --- | --- | --- | --- | --- | --- | --- |
|  | **lnL** | **ω0** | **P0** | **ω2** | **P1** | **2λ** | **df** | **P value** |
| **A2-15** | -3129.63 | 0.050 | 0.864 | 2.300 | 0.025 | 0.180 | 1 | 0.672 |
| **A4-5-6** | -3833.07 | 0.014 | 0.945 | 1.000 | 0.055 | 1.734 | 1 | 0.188 |
| **A7-8-9** | -3515.39 | 0.042 | 0.872 | 3.568 | 0.0286 | 1.549 | 1 | 0.213 |
| **A12-13** | -2608.37 | 0.018 | 0.963 | 7.897 | 0.037 | 0 | 1 | 1 |
| **A14-30** | -3127.56 | 0.018 | 0.906 | 1.000 | 0.019 | 0 | 1 | 1 |
| **A18-22** | -3198.51 | 0.015 | 0.876 | 999.00 | 0.032 | 2.371 | 1 | 0.1236 |
| **A23-24-25** | -3242.23 | 0.026 | 0.733 | 1.804 | 0.046 | 0.044 | 1 | 0.8342 |
| **A28-37** | -2369.35 | 0.024 | 0.768 | 1.000 | 0.060 | 0 | 1 | 1 |
| **A33-36** | -3103.36 | 0.021 | 0.864 | 1.000 | 0.038 | 0 | 1 | 1 |
| **A34-35** | -4821.53 | 0.094 | 0.792 | 238.604 | 0.134 | 1.085 | 1 | 0.2975 |
| **A39-40** | -3525.97 | 0.048 | 0.810 | 1.000 | 0.014 | 0 | 1 | 1 |
| **A45-47** | -3572.450 | 0.037 | 0.923 | 1.000 | 0.078 | 0 | 1 | 1 |

**Additional file 3**

**Likelihood scores, model parameters, and likelihood-ratio test results for branch-site model analyses.** A indicates the orthologous sequences of member of SLC25. The index in SLC25 (the appellation of MCF in human) was used to denote the corresponding orthologous sequences, such as A7, 8, 9 (representing the orthologous sequences of UCP1, 2, 3, respectively). We used a branch-site test of positive selection [44] to construct a likelihood ratio test between Model A with ω_2_≧1( alternative model,) and Model A1 with ω_2_= 1 fixed (null model). The test was done by comparing the difference of likelihood values 2λ to a χ2 distribution of 1 degree of freedom. The critical values of 2λ at the 5% and 1% level are 2.71 and 5.41, respectively [49].
